# Effect of heat processing on selected grain amaranth physicochemical properties

**DOI:** 10.1002/fsn3.75

**Published:** 2013-11-15

**Authors:** John H Muyonga, Brian Andabati, Geoffrey Ssepuuya

**Affiliations:** School of Food Technology, Nutrition & Bioengineering, Makerere UniversityP.O. Box 7062, Kampala, Uganda

**Keywords:** Antioxidant activity, flavonoids, grain amaranth, pasting properties, phenolics, protein digestibility

## Abstract

Grain amaranth is a pseudocereal with unique agricultural, nutritional, and functional properties. This study was undertaken to determine the effect of different heat-processing methods on physicochemical and nutraceutical properties in two main grain amaranth species, of *Amaranthus hypochondriacus* L. and *Amaranthus cruentus* L. Grains were prepared by roasting and popping, milled and analyzed for changes in in vitro protein digestibility, gruel viscosity, pasting characteristics, antioxidant activity, flavonoids, and total phenolics. In vitro protein digestibility was determined using the pepsin-pancreatin enzyme system. Viscosity and pasting characteristics of samples were determined using a Brookfield Viscometer and a Rapid Visco Analyzer, respectively. The grain methanol extracts were analysed for phenolics using spectrophotometry while antioxidant activity was determined using the DPPH (2,2-diphenyl-1-picrylhydrazyl) method. Heat treatment led to a reduction in protein digestibility, the effect being higher in popped than in roasted samples. Viscosities for roasted grain amaranth gruels were significantly higher than those obtained from raw and popped grain amaranth gruels. The results for pasting properties were consistent with the results for viscosity. In both *A. hypochondriacus* L. and *A. cruentus* L., the order of the viscosity values was roasted>raw>popped. The viscosities were also generally lower for *A. cruentus* L. compared to *A. hypochondriacus* L. Raw samples for both *A. hypochondriacus* L. and *A. cruentus* L. did not significantly differ in total phenolic content (TPC), total flavonoid content (TFC), and total antioxidant activity values. Thermal processing led to an increase in TFC and antioxidant activity. However, TPC of heat-processed samples remained unchanged. From the results, it can be concluded that heat treatment enhances antioxidant activity of grain amaranth and causes rheological changes dependent on the nature of heat treatment.

## Introduction

Grain amaranth has the potential to contribute to improvement in nutrition of populations, especially in developing countries, because of its unique agricultural, nutritional, and functional properties. It is fast-growing, high-yielding, stress-resistant, nutritious, and has nutraceutical properties. Grain amaranth is rich in proteins, lipids, energy, and fiber (Muyonga et al. 2008). Grain amaranth protein is of superior amino acid profile compared to proteins found in most other plant foods. Amaranth grains contain twice the level of calcium in milk, five times the level of iron in wheat, higher sodium, potassium, and vitamins A, E, C, and folic acid than cereal grains (Becker et al. 1981).

Grain amaranth has been shown to exhibit antioxidant activity and this has been attributed to its content of polyphenols, anthocyanins, flavonoids, and tocopherols (Klimczak et al. 2002; Escudero et al. 2011). Phenolic content of grain amaranth varies between species and may be affected by environmental conditions (Escudero et al. 2011). The antioxidant activity of phenolics is associated with inhibition of lipid peroxidation (Charanjit et al. 2009). Animal models have shown protective effects of grain amaranth against serum and liver intoxication (López et al. 2011). Amaranth oil has been shown, in animal studies, to lower total serum triglycerides and levels of low-density lipoproteins (Berger et al. 2003; Escudero et al. 2006; Martirosyan et al. 2007). Consumption of grain amaranth has been associated with health benefits in humans, including recovery of severely malnourished children and increase in the body mass index of people formerly wasted by HIV/AIDS (Tagwira et al. 2006).

A variety of heat-processing methods are applied to grain amaranth, in preparation for consumption. Heat processing affects the level of phytochemicals (Xu et al. 2007), antioxidant activity (Xu et al. 2007; Queiroz et al. 2009), functional properties (Muyonga et al. 2001), and nutritional value (Rehman and Shah 2005) of foods. Rheological properties of grain amaranth have also been shown to vary among species (Kong et al. 2009). The aim of this study was to investigate the effect of different heat-processing methods commonly applied to grain amaranth on the protein digestibility, rheology, phenolic content, and antioxidant activity of two grain amaranth species.

## Material and Methods

### Grain amaranth

Amaranth (*A. hypochondriacus* L. and *A. cruentus* L.) grains were procured from farmers in Kamuli district, Uganda, who had previously been supplied with seeds for the two species by Makerere University School of Agricultural Sciences. Grains were color sorted to ensure sample purity.

### Grain processing

Grains of the two types were separately processed by roasting and popping. Roasting was performed in a Gallenkamp oven 282A (Fistreem International, Leicestershire, U.K.). About 1 kg of dry amaranth grain was spread uniformly on a baking tray of ∼0.3 × 0.6 m in size. The amaranth was roasted at 200°C for 8 min after which it was cooled and later milled into flour.

Amaranth was popped by heating on an aluminum pan using an Ariston K3G2/G gas cooker (Ariston Appliances, Boston, MA) set to maximum heat. A handful of dry grain amaranth (∼100 g) of grain was placed on the pan at a time and heated for about 1–2 min, while stirring using a wooden ladle. The grains began to pop after heating for ∼30 sec. Heating was continued until almost all the grain turned whitish. The total heating time per batch was ∼90 sec. On completion of popping, the popped grains were passed over a 1-mm mesh to separate the popped from those that had not popped grains. The grains that had not popped passed through the screen while the popped grains did not.

Raw grains of the two types as well as the grains processed by the different heat treatment methods were ground using a Waring Blender HGB55E (Waring Blender Co., McConnellsburg, PA) and passed through a Retsch 500-*μ*m sieve (Haan, Germany). The resulting flour was stored (for ≤30 days) in airtight glass jar at room temperature (∼25°C) until analysis was undertaken.

### Determination of physicochemical properties

#### Protein digestibility

In vitro protein digestibility was determined using the pepsin–pancreatin enzyme system (Saunders et al. 1973; Chavan et al. 2001) with minor modifications. About 1 g of sample was suspended in 60 mL of 0.1 mol\L HCl containing 6 mg of pepsin, followed by gentle shaking for 15 min at 37°C. The resulting solution was then neutralized with 0.5 mol\L NaOH and treated with 16 mg of pancreatin from porcine pancreas (activity equivalent to 4× US pharmacopeia) in 30 mL of phosphate buffer (0.1 mol\L, pH 8.0). The mixture was then shaken for 24 h at 37°C in a water bath shaker. The undigested solid was separated by filtration using glass wool (of known weight) under suction from a vacuum pump and washed twice with 10 mL distilled water. The protein content in the undigested solid and initial protein content of both cooked and raw samples was determined using the Kjeldahl method. Digestibility was calculated using the formula:


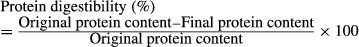


#### Viscosity

Viscosity was determined using a Brookfield DV-II+Pro Viscometer LVDV-11+P (Brookfield Engineering Laboratories, Inc., Middleboro, MA). For all the flour samples, the same mass to volume ratio (1:10) was used to prepare porridge. Typically, 50 ± 10 g of flour and 500 ± 100 mL of water were used. The mixture of flour in water was boiled for 20 before pre-boiled hot water was added to attain the total water volume required to make 6% or 9% solid content. Boiling was continued for an additional 5 min. The beakers and their contents were then placed in a water bath maintained at 60°C until the gruels cooled to a temperature of 60–62°C. Upon recording the temperature of 60–62°C, the gruel were analysed for viscosity using spindle #4 and at spindle speed of 30 revolutions per min. Viscosity readings were taken 60 sec after turning off the rotor.

#### Pasting properties

Pasting characteristics of the flours were determined using a Rapid Visco Analyzer RVA-4 (Newport Scientific Pty. Ltd., Warriewood, Australia). The flour suspensions (6.72 g in 25.28 mL H_2_O) corrected to 14% humidity base were exposed to the following time/temperature sequence: 50°C for 1 min, heating from 50°C to 95°C at 12.16°C/min, maintained at 95°C for 2.5 min, and cooled from 95°C to 50°C at 11.84°C/min rate. The apparent viscosity was expressed in rapid visco units. Peak viscosity, trough, breakdown, final viscosity, set back, peak time, and pasting temperature were recorded.

### Determination of bioactive compounds and antioxidant activity

#### Extraction of flavonoids and phenolic compounds

Methanol extracts were obtained from all samples (Makkar 2000). Briefly, ∼0.1 g of flour sample was extracted for 30 min with 5 mL of methanol:water (50:50 v/v) mixture at room temperature, while intermittently shaking. The extract was cooled by keeping the extract tube in a freezer for 10 min and then centrifuged at 3000 *g* for 10 min using a FisherScientific centrifuge 225 (Fisher Scientific, Leicestershire, U.K.). The supernatant was recovered and the pellet re-extracted for 45 min under the same conditions. Finally, the two supernatants were pooled and used for total antioxidant activity, total phenolic content (TPC), and total flavonoid content (TFC).

#### Antioxidant activity

The antioxidant activity of the methanol extracts was estimated using the DPPH (1,1-diphenyl-2-pycrylhydrazyl) free radical-scavenging assay (Kim et al. 2002). To 2.95 mL of freshly prepared absolute methanol solution of 100 *μ*mol\L of DPPH, 50 *μ*L of the sample extract or control (50% [v/v] methanol) was added. The mixture was shaken and allowed to stand at room temperature in the dark for 30 min. The absorbance of the resulting solution was measured at 517 nm against a blank (absolute methanol). The free radical-scavenging activity was calculated as follows:


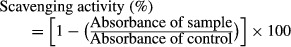


A standard of ascorbic acid was run using several concentrations ranging from 0.002 to 0.1 mg/mL. A standard curve was constructed by plotting the percentage of free radical-scavenging activity of ascorbic acid versus its concentration (*R*^2^ = 0.992). The final result was expressed as mg vitamin C equivalent per 1 g dry weight (mg VCE/g dw).

#### Determination of total phenolic content

Total phenolic content was determined using spectrophotometry (Makkar 2000). To a sample of 100 *μ*L, distilled water was added to make the quantity 0.5 mL. This was followed by the addition of 0.25 mL of Folin-Ciocalteu reagent (1 N) and 1.25 mL of sodium carbonate (20%). After 40 min at room temperature, the absorbance at 725 nm was read on a GENESYS spectrophotometer 10 ultraviolet (Thermo Electron Corporation, Marietta, OH) against a blank that contained methanol instead of a sample. The calibration curve was constructed within the concentration range 0.025–0.225 mg/mL (*R*^2^ = 0.999). The TPC values were expressed in milligrams of gallic acid equivalents/gram dry weight (mg GAE/g dw) of plant material using equation:


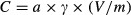


where *C* is the total amount of phenolic compounds (mg GAE/g dw sample), *a* is the dilution number, *γ* is the concentration obtained from the calibration curve (mg/mL), *V* is the volume of aqueous methanol used for extraction (mL), and *m* is the weight of dry plant material (g).

#### Determination of total flavonoid content

Total flavonoid content was measured using a colorimetric assay (Muanda et al. 2011). A quantity of 250 *μ*L standard solution of catechin at different concentrations or appropriately diluted samples was added to a 10 mL volumetric flask containing 1 mL of double distilled waters (ddH_2_O). At time 0 min, 75 *μ*L of NaNO_2_ (5%) was added to the flask. After 5 min, 75 *μ*L of AlCl_3_ (10%) was added. At 6 min, 500 *μ*L of NaOH (1N) was added to the mixture. The solution was then diluted by adding 2.5 mL double-distilled H_2_O and mixed thoroughly. The absorbance of the pink mixture was determined at 510 nm against a blank that contained distilled water instead of a sample. The calibration curve was constructed within the concentration range 0.025–0.225 mg/mL (*R*^2^ = 0.999). The TFC values for samples were expressed as milligrams of catechin equivalents/gram dry weight (mg CE/g dw) of plant material using the equation:


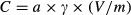


where *C* is the total amount of phenolic compounds (mg GAE/g dw sample), *a* is the dilution number, *γ* is the concentration obtained from the calibration curve (mg/mL), *V* is the volume of aqueous methanol used for extraction (mL), and *m* is the weight of dry plant material (g).

### Statistical analysis

Data for all parameters corresponding to different treatments were subjected to analysis of variance (ANOVA) at *α *= 0.05 using the SPSS (version 16, SPSS Inc., Chicago, IL). Means were separated using least significance difference (LSD). All analytical measurements were performed in triplicate.

## Results and Discussion

### Protein digestibility

The protein content was found to be 12.37 ± 0.71% and 13.04 ± 0.98%, respectively, for the *A. cruentus* L. and *A. hypochondriacus* L. variety. Protein digestibility for *A. cruentus* L. (73.85%) grain amaranth and *A. hypochondriacus* L. (71.93%) were not significantly different. In vitro digestibility of 61–76% has previously been reported for raw grain amaranth proteins (Correa et al. 1986). Heat treatments led to a reduction in protein digestibility (Table 1). This is in agreement with an earlier work (Písaŕíkova et al. 2005) that reported a reduction in in vitro protein digestibility from 68.1 to 50.6 as a result of popping. The reduction in protein digestibility in this study was higher in popped than in roasted samples. The reduction in protein digestibility resulting from heat processing of grain amaranth might be attributed to amino acid degradation, formation of intramolecular disulfide bonds and Maillard reaction, changes associated with dry heat processing (Hurrell et al. 1976; Hsu et al. 1977; Nestares et al. 1993). The lower digestibility for popped seeds as compared to roasted ones point to more pronounced protein changes popping. This is not surprising as popping temperatures tend to be higher than those registered during roasting. The in vitro protein digestibility values recorded from grain amaranth were higher than reported digestibility values for whole raw maize (66.6%) and sorghum (55.8–59.1%) (Duodu et al. 2002). Grain amaranth proteins therefore seem to exhibit higher digestibility than these cereals. Heat treatment has been reported to cause reduction in protein digestibility for sorghum and increased digestibility for maize (Duodu et al. 2002). The nature of the change in protein digestibility resulting from heat treatment seems to relate partly to the extent of formation of complexes between proteins and other grain components and the level of matrix disintegration, which impacts the access of proteolytic enzymes to protein bodies.

**Table 1:** Protein digestibility (%) of raw, roasted, and popped grain amaranth

Treatment	Protein digestibility (%)
*A. cruentus* L. raw	73.85 ± 2.11^a^
*A. cruentus* L. roasted	63.34 ± 1.23^b^
*A. cruentus* L. popped	52.81 ± 1.34^c^
*A. hypochondriacus* L. raw	71.93 ± 3.03^a^
*A. hypochondriacus* L. roasted	60.60 ± 2.23^b^
*A. hypochondriacus* L. popped	50.51 ± 1.44^c^

Data are expressed as means ± SE for triplicate experiments. Means with same superscript are not significantly different.

### Viscosity

Samples of *A. hypochondriacus* L. consistently exhibited higher viscosity compared with those for *A. cruentus* L. (Table 2). Viscosity differences among grain amaranth cultivars have been shown to correlate positively with amylose content (Kong et al. 2009). Studies on starch from different sources showed that amylose content and amylopectin branch chain length distribution predominantly affect starch pasting properties (Jane et al. 1999). The difference in pasting properties of the two grain amaranth species in this study may therefore reflect differences in amylose content and/or nature of amylopectin in their starches. Popped samples exhibited much lower viscosity than raw samples (Table 2). This can be attributed to pregelatinization of starch due to heat treatment. On the contrary, roasted samples showed significantly higher viscosity than raw and popped samples. During heating, starch granules may disintegrate, becoming more susceptible to hydration which is associated with high viscosity (Lai 2001). Leached amylose may also associate resulting in limited rehydration potential and lower peak viscosity. Heat pretreatment may therefore increase or reduce paste viscosity. In this case, the observed high viscosity for roasted grain amaranth may be attributed to disintegration of starch granules, increasing their susceptibility to hydration while the low viscosity of flour from popped grain seems to arise from the association of amylose, possibly because of the extreme dehydration impact of popping. The viscosity values show that the drinking consistency (∼3000 cP) is achieved at a flour rate of between 6% and 9% for raw grain amaranth. Much higher rates are required in the case of popped amaranth while much lower rates would be required for roasted grain.

**Table 2:** Viscosity of 6% and 9% gruels of *Amaranthus hypochondriacus* L. and *A. cruentus* L

Amaranth variety		*A. hypochondriacus* L.	*A. cruentus* L.
Treatment	% flour	Viscosity, cP at 40°C	Viscosity, cP at 40°C
Raw	6	2240 ± 34.2^d,1^	1999 ± 30.1^d,2^
	9	8798 ± 54.6^c,1^	6299 ± 81.1^c,2^
Roasted	6	31643 ± 712.1^b,1^	17496 ± 621.1^b,2^
	9	37268 ± 862.5^a,1^	31593 ± 822.8^a,2^
Popped	6	322.9 ± 11.7^f,1^	213.0 ± 16.1^f,2^
	9	834 ± 19.0^e,1^	625.9 ± 41.1^e,2^

Data are expressed as means ± SE for triplicate experiments. Means within a column with the same superscript are not significantly different. Means within the same row with the same numeral are not significantly different.

### Pasting properties

**Figure 1:**
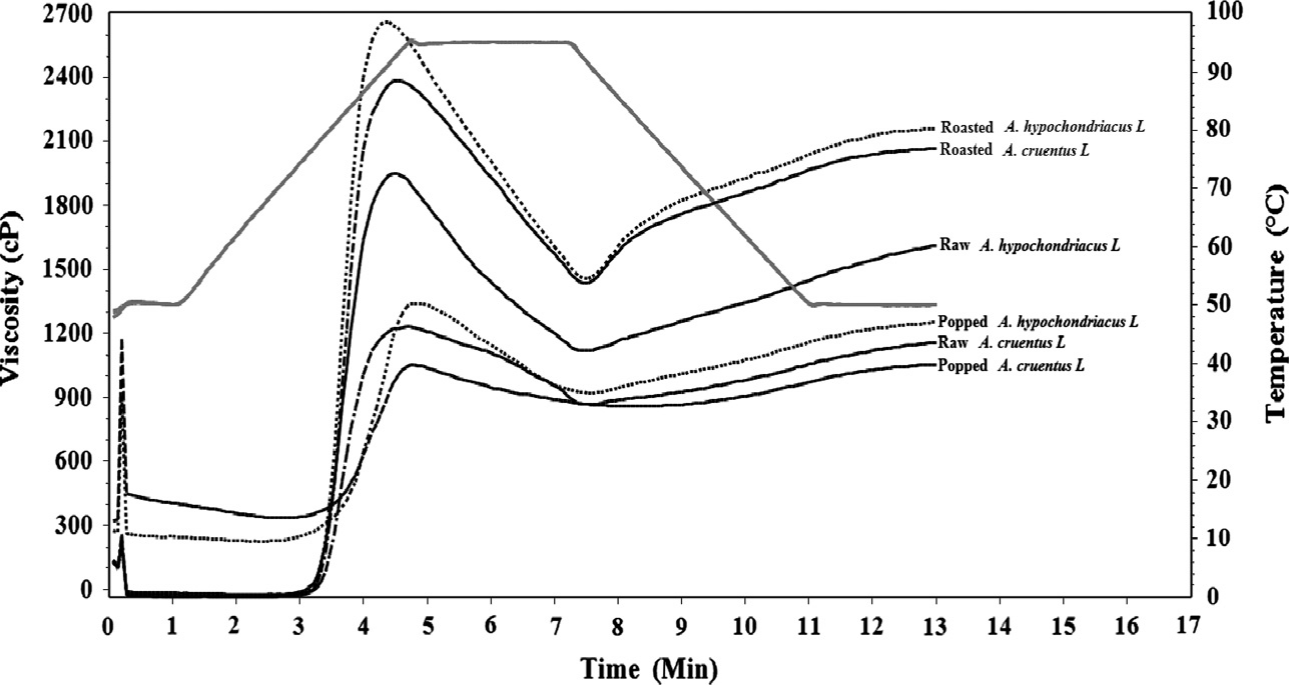
The RVA profiles of raw, roasted, and popped *Amaranthus hypochondriacus* L. and *A. cruentus* L.

The results for pasting properties were consistent with the results for viscosity. In both *A. hypochondriacus* L. and *A. cruentus* L., the order of viscosity values was roasted>raw>popped (Fig. 1). The viscosities were also generally lower for *A. cruentus* L. compared to *A. hypochondriacus* L. Amylose content has been established as a key determinant of pasting viscosity for grain amaranth cultivars (Kong et al. 2009). The observed differences in pasting temperature between species seem to suggest that the two differ in their amylose content. A study of 15 grain amaranth cultivars grown in China (11 belong to *A. cruentus* L. and 2 to *A. hypochondriacus* L.) revealed amylose content of 5.4–5.8% for *A. hypochondriacus* L. and 4.7–12.5% for *A. cruentus* L. (Kong et al. 2009). The pasting temperatures for raw *A. hypochondriacus* L. and *A. cruentus* L. in this study were found to be 75.9°C and 77.6°C, respectively (Table 3). Pasting temperatures of 63.4–74°C have been reported for grain amaranth from previous studies (Lai 2001; Kong et al. 2009). The effect of roasting and popping on pasting viscosity was generally similar to that reported above for viscosity. High pasting viscosity was associated with high breakdown viscosity, high final viscosity, high setback, low pasting time, and temperature. A similar trend was reported when comparing pasting properties of different grain amaranth cultivars (Kong et al. 2009). This trend can be explained by the fact that all these attributes are dependent on the pace and level of starch granule disintegration. Samples which register more extensive granule disintegration seem also likely to exhibit a high extent of retrogradation reflected in the values for setback.

**Table 3:** Pasting properties of raw, roasted, and popped *Amaranthus hypochondriacus* and *A. cruentus* L. amaranth

Amaranth variety	Pasting viscosity	Breakdown viscosity	Final viscosity	Setback	Pasting time (min)	Pasting temperature (°C)
Raw *A. cruentus* L.	1222.5 ± 12.0^e^	258 ± 7.07^e^	1159.5 ± 7.8^e^	207 ± 4.2^e^	4.68 ± 0.01^c^	77.63 ± 0.1^c^
Roasted *A. cruentus* L.	2405.5 ± 33.2^b^	963.5 ± 26.16^b^	2078.5 ± 14.9^b^	636.5 ± 7.8^b^	4.57 ± 0.05^d^	76.73 ± 0.1^d^
Popped *A. cruentus* L.	1006.0 ± 66.5^f^	176.5 ± 28.99^f^	1026 ± 41.0^f^	196.5 ± 3.5^f^	4.84 ± 0.05^a^	81.23 ± 0.5^a^
Raw *A. hypochondriacus* L.	1963.5 ± 21.9^c^	847 ± 24.04^c^	1625 ± 21.2^c^	508.5 ± 23.3^e^	4.47 ± 0.00^e^	75.93 ± 0.1^e^
Roasted *A. hypochondriacus* L.	2711.5 ± 75.7^a^	1213 ± 15.55^a^	2204 ± 63.6^a^	705.5 ± 3.5^a^	4.33 ± 0.00^f^	75.95 ± 0.1^f^
Popped *A. hypochondriacus* L.	1359.5 ± 26.2^d^	417.5 ± 3.54^d^	1255.5 ± 7.8^d^	313.5 ± 21.9^d^	4.77 ± 0.05^b^	80.03 ± 0.1^b^

Data are expressed as means ± SE for triplicate experiments. Means within a column with the same superscript are not significantly different.

### Total phenolic content

No significant difference in TPC was observed between raw and roasted or popped grain for both *A. cruentus* L. and *A. hypochondriacus* L. (Table 4). Earlier studies have reported conflicting results with respect to the effect of heat on total phenolics. Heat has been reported to cause reduction in TPC of roasted and popped grain amaranth (Yanez et al. 1986). This reduction may be attributed to thermal degradation due to the processing conditions used in the study (Griffith and Castell-Perez 1998). Roasting of sesame seeds at 200°C for 20 min, on the other hand, was found to significantly increase TPC (Devi et al. 2011). Increased phenolic content has been reported for tomatoes treated at 88°C for 30 min (Choi et al. 2006). The increased phenolic content of thermally processed foods can be attributed to the heat-induced release of more bound phenolics (Dewanto et al. 2002; Jannat et al. 2010). Therefore, the results of this study may be attributed to the heat-associated increase in phenolic compounds offsetting their degradation by heat.

**Table 4:** Total phenolic content, total flavonoid content, and DPPH for *Amaranthus hypochondriacus* L. and *A. cruentus* L

Material tested	Total phenolic content (mg GAE/g dw)	Total flavonoid content (mg CE/g dw)	Total antioxidant activity (mg VCE/g dw)
Raw *A. cruentus* L.	3.63 ± 0.08^ab^	0.54 ± 0.13^b^	0.24 ± 0.12^bc^
Roasted *A. cruentus* L.	3.93 ± 0.22^a^	1.06 ± 0.18^a^	0.56 ± 0.07^a^
Popped *A. cruentus* L.	3.41 ± 0.32^ab^	0.93 ± 0.16^a^	0.31 ± 0.13^bc^
Raw *A. hypochondriacus* L.	3.34 ± 0.22^b^	0.47 ± 0.09^b^	0.09 ± 0.01^c^
Roasted *A. hypochondriacus* L.	3.70 ± 0.33^ab^	0.54 ± 0.07^b^	0.33 ± 0.08^b^
Popped *A. hypochondriacus* L.	2.99 ± 0.29^b^	0.78 ± 0.11^a^	0.13 ± 0.07^c^

Data are expressed as means ± SE for triplicate experiments. Means within a column with the same superscript are not significantly different.

### Flavonoid content

Heat treatment generally led to an increase in the flavonoid content in grain amaranth. An increase in flavonoid content has also been reported following heating of Shiitake (*Lentinus edodes*) mushroom (Choi et al. 2006). This was attributed to enhanced extractability of bound flavonoid compounds resulting from heat-induced disruption of the plant cell wall. Heat-induced increase in flavonoid content has also been associated with deactivation of endogenous oxidative enzymes, thereby preventing enzymatic oxidation which causes loss of the antioxidant compounds in the raw plant materials (Jeong et al. 2004; Jannat et al. 2010).

### Antioxidant activity

Roasting resulted in a significant increase in antioxidant activity of both *A. hypochondriacus* L. and *A. cruentus* L. Roasting has been previously reported to increase the antioxidant activity of sesame seeds (Devi et al. 2011). Much of the antioxidant activity of plant materials is attributable to flavonoids and other phenolics (Kahkonen et al. 1999; Nicoli et al. 1999). Therefore, the increase in antioxidant activity might be due to the observed increase in total flavonoids (Table 4). Popping on the other hand did not cause a significant change in antioxidant activity of both *A. hypochondriacus* L. and *A. cruentus* L. This trend may be attributed to the negative and positive effect of heat on different phenolic compounds.

## Conclusion

The two varieties of grain amaranth (*A. hypochondriacus* L. and *A. cruentus* L.) studied differ in physicochemical properties, with *A. cruentus* L. exhibiting higher protein content and higher antioxidant activity than *A. hypochondriacus* L. and *A. hypochondriacus* L. exhibiting higher viscosity. When exposed to dry heat processes typically used to prepare grain amaranth, both protein digestibility and antioxidant activity are affected. Popping has a higher negative impact on protein digestibility while roasting is more damaging to the antioxidant activity. Heat processing also leads to change in viscosity and pasting behavior of grain amaranth. The results show that roasting would be preferred in the production of flour to be used as a thickening agent or for low-calorie gruels. On the other hand, popping is suitable for the production of flour for high nutrient density gruels.
